# Grand Challenges in Environmental Psychology

**DOI:** 10.3389/fpsyg.2016.00583

**Published:** 2016-04-25

**Authors:** Patrik Sörqvist

**Affiliations:** Department of Building, Energy and Environmental Engineering, University of GävleGävle, Sweden

**Keywords:** environmental psychology, grand challenges, sociophysical environment, built environment, natural environment

Environmental psychology is the subdiscipline of psychological science that deals with psychological processes engaged in encounters between people and the built and natural environment (Stern, 2000). It covers all aspects of human behavior and mental life in relation to the sociophysical environment, whether considered as ambient environmental factors (e.g., noise, temperature, lighting), specific behavior settings (e.g., schools, offices, hospitals), the basic infrastructure of everyday life (e.g., energy and transportation systems), or in a broader sense, with regard to landscape and the relationship between built and natural aspects of human environments. Human behavior and mental life include, but are not limited to, perception and cognition, emotion, stress and mental fatigue, decision making, and social interactions, as manifest in covert and overt behavior. In short, environmental psychology is concerned with the facts of bi-directional influence in people-environment interactions; it considers how the sociophysical environment influences people and how people influence the environment (Gärling, 2014).

Global climate change is currently one of society's grand challenges (American Psychological Association, 2008; Hansen et al., 2013). Psychology cannot by itself slow or halt global warming, but it can explain why people sometimes engage in pro-environmental behavior that can mitigate climate change and it can help citizens overcome the psychological barriers of sustainable behavior (Gifford, 2011). A grand challenge for the environmental psychologist is to study, explain and predict how people's behavior can be changed to promote environmentally sustainable behavior (Vlek and Stag, 2007; Kaiser et al., 2013), environmentalism (Dietz et al., 1998) and conservation (Cialdini, 2003). One approach to this grand challenge involves the use of normative messages (Cialdini, 2003), framings (Hurlstone et al., 2014), social norms (Clark et al., 2008; Bertoldo et al., 2013) and educational programs (Ernst and Theimer, 2011) to reduce people's environmental footprint through communication and information interventions. This view aligns with a social psychological tradition with an emphasis on the study of how personality traits and attitudes shape behavior. Another approach—largely neglected in contemporary environmental psychology (Gärling, 2014)—focuses on how the environment itself should be built and modified to support and even require more sustainable human behavior (Johansson et al., 2016). This latter view stems from a behavioral and ecological approach to human functioning and focuses on how the environment shapes behavior (Scott, 2005). While the social psychological view has unquestionable merits, I envision a practicable way toward scientific breakthrough is to reintroduce this classic, ecological approach in environmental psychology and apply it to the modern problems of society.

The current anthropogenic global warming is coupled with an exponential human population growth that is placing tremendous demands on agricultural and natural resources (Foley et al., 2011). Environmental psychologists will play an important role in providing society with needed insights in several areas, including how to handle the social dilemmas of sharing resources in sustainable ways (Biel and Gärling, 1995; Anderies et al., 2013), how to implement techniques to mitigate the effects of increased energy demand (Nilsson et al., 2015) and how to understand the psychological consequences of scarcity (Griskevicius et al., 2013). One way to deal with the consequences of the population growth is to build megacities with high residential densities, as there are gains in energy and transportation efficiency to be made which can help to mitigate the negative effects of human activity on the environment (Kennedy et al., 2015). A major challenge with this development, however, is that such urbanization can lead to built environments that many people will find unlivable. A likely scenario is that the availability and access to natural environments are reduced, whereby people risk the loss of restorative opportunities supported by interaction with nature (Hartig et al., 2014), at the same time that stressful experiences of crowding (Lederbogen et al., 2011), noise (Basner et al., 2015) and air pollution become more common (Folberth et al., 2015). Furthermore, large cities must allow for a emergency services and other functions to work around the clock. People's dependency on artificial lighting will probably increase, and consequently so will also the usurpation of energy resources (Maleetipwan-Mattsson et al., 2016). The environmental psychologist faces the need to understand the effects of new environmental, work and living conditions for the human being, including land- (Hagerhall et al., 2004) and soundscapes (Nilsson and Berglund, 2006), schools (Clark et al., 2013), offices (Hongisto et al., 2016), and hospitals (Ulrich et al., 2008). The grand challenge is to act proactively, to study the general laws of how ambient factors and behavioral settings influence humans, and to develop models—well-grounded in theory and sound conceptualization—to predict the effects of alternative future environments.

To this end, environmental psychology will have to confront a range of theoretical, methodological and conceptual challenges. These include applying knowledge from cognitive psychology—on memory, attention, perception and performance (Sörqvist, 2010, 2015)—to classic questions in environmental psychology, such as how natural environments can facilitate restoration from attentional fatigue (Kaplan, 1995); how brain imaging methods can be employed to address how the human brain interacts with the built and natural environment (Lederbogen et al., 2011; Lambert et al., 2015); and how evolutionary perspectives can inform environmental design (Joye, 2007). The challenges also include understanding the limits of these perspectives (Joye and van den Berg, 2011) and questioning commonly accepted claims; for example, asking whether the natural environment always has an advantage over the built environment with regard to its cognitive effects. I envision the specialty section on environmental psychology to be a place for researchers to express and discuss empirical findings, opinions, theories and hypotheses channeled through publication forms not available in any other environmental psychology outlet. The Frontiers' publication platform with article types like *opinion, methods* and *hypothesis and theory* provides an excellent opportunity for scientific debate about the fundamental mechanisms of psychological phenomena which I encourage authors to exploit for empirical, methodological, theoretical and conceptual development of our field.

## Author contributions

The author confirms being the sole contributor of this work and approved it for publication.

### Conflict of interest statement

The author declares that the research was conducted in the absence of any commercial or financial relationships that could be construed as a potential conflict of interest.
